# CT-Based Radiomics Analysis for Noninvasive Prediction of Perineural Invasion of Perihilar Cholangiocarcinoma

**DOI:** 10.3389/fonc.2022.900478

**Published:** 2022-06-20

**Authors:** Peng-Chao Zhan, Pei-jie Lyu, Zhen Li, Xing Liu, Hui-Xia Wang, Na-Na Liu, Yuyuan Zhang, Wenpeng Huang, Yan Chen, Jian-bo Gao

**Affiliations:** ^1^ Department of Radiology, The First Affiliated Hospital of Zhengzhou University, Zhengzhou, China; ^2^ Henan Key Laboratory of Imaging Diagnosis and Treatment for Digestive System Tumor, Zhengzhou, China; ^3^ Department of Interventional Radiology, The First Affiliated Hospital of Zhengzhou University, Zhengzhou, China

**Keywords:** perihilar cholangiocarcinoma, perineural invasion, radiomics, CT, nomogram

## Abstract

**Purpose:**

The study aimed to construct and evaluate a CT-Based radiomics model for noninvasive detecting perineural invasion (PNI) of perihilar cholangiocarcinoma (pCCA) preoperatively.

**Materials and Methods:**

From February 2012 to October 2021, a total of 161 patients with pCCA who underwent resection were retrospectively enrolled in this study. Patients were allocated into the training cohort and the validation cohort according to the diagnostic time. Venous phase images of contrast-enhanced CT were used for radiomics analysis. The intraclass correlation efficient (ICC), the correlation analysis, and the least absolute shrinkage and selection operator (LASSO) regression were applied to select radiomics features and built radiomics signature. Logistic regression analyses were performed to establish a clinical model, a radiomics model, and a combined model. The performance of the predictive models was measured by area under the receiver operating characteristic curve (AUC), and pairwise ROC comparisons between models were tested using the Delong method. Finally, the model with the best performance was presented as a nomogram, and its calibration and clinical usefulness were assessed.

**Results:**

Finally, 15 radiomics features were selected to build a radiomics signature, and three models were developed through logistic regression. In the training cohort, the combined model showed a higher predictive capability (AUC = 0.950) than the radiomics model and the clinical model (AUC: radiomics = 0.914, clinical = 0.756). However, in the validation cohort, the AUC of the radiomics model (AUC = 0.885) was significantly higher than the other two models (AUC: combined = 0.791, clinical = 0.567). After comprehensive consideration, the radiomics model was chosen to develop the nomogram. The calibration curve and decision curve analysis (DCA) suggested that the nomogram had a good consistency and clinical utility.

**Conclusion:**

We developed a CT-based radiomics model with good performance to noninvasively predict PNI of pCCA preoperatively.

## Introduction

Cholangiocarcinoma (CCA) is a highly lethal malignancy originating from the biliary epithelium, accounting for about 3% of all gastrointestinal system malignancies (1, 2). According to the anatomical location, CCA is divided into three subtypes, and perihilar cholangiocarcinoma (pCCA) represents the most common type of CCA (3). PCCA carries a poor prognosis with a median overall survival (OS) of 5-10 months (4). Surgery resection with an R0 margin can significantly prolong OS and provide a chance to cure pCCA. However, only 13% to 32% of pCCA patients undergo surgical resection and the recurrence rate within one year was greater than 50% (4, 5).

PNI is considered one of the histological features of biliary tract tumors, and it has a high prevalence in biliary tract tumors ranging from 56% to 88% (6). Previous studies reported that PNI was an important risk factor associated with poor prognosis and low overall survival time of malignant tumors (7–9). In pCCA, the 5-year overall survival of patients without PNI is 63.7%, whereas that of patients with PNI is 34.9% (10). In addition, PNI was regarded as an independent risk factor for R0 resection and presented a high recurrence rate (11, 12). At present, several studies have made a progress in the molecular mechanism of PNI, which may block the occurrence of PNI and lead to specific tumor treatments (6, 13, 14). Therefore, the diagnosis of PNI is essential for determining the treatment approach and predicting the prognosis of patients with pCCA.

Multidetector computed tomography (MDCT) and magnetic resonance imaging (MRI) are the most commonly used imaging modalities for the diagnosis and evaluation of pCCA (15). However, they are of limited value for the detection of PNI. Currently, PNI can only be confirmed by pathological examination of surgical specimens, and it is invasive and can only be performed after resection. Developing an appropriate and convenient method for the noninvasive prediction of PNI is urgently required.

Radiomics is a new rapidly developing technology that can extract numerous quantitative features from medical images, and these imaging features may be informative for disease diagnosis, prognosis, and treatment response (16–18). Some studies have indicated the potential value of radiomics for the preoperative prediction of PNI in specific tumors (19–25). Nevertheless, we have not yet found any study that focuses on CT-based radiomics analysis for the noninvasive prediction of PNI of pCCA. Therefore, the goal of our study was to construct and evaluate a CT-Based radiomics model for noninvasive detecting PNI of pCCA.

## Materials and Methods

### Patient Characteristics

This retrospective, single-center study was approved by the Ethical Committee of the First Affiliated Hospital of Zhengzhou University (2021-KY-0778-001), and the requirement for informed consent was waived.

**Figure 1:**
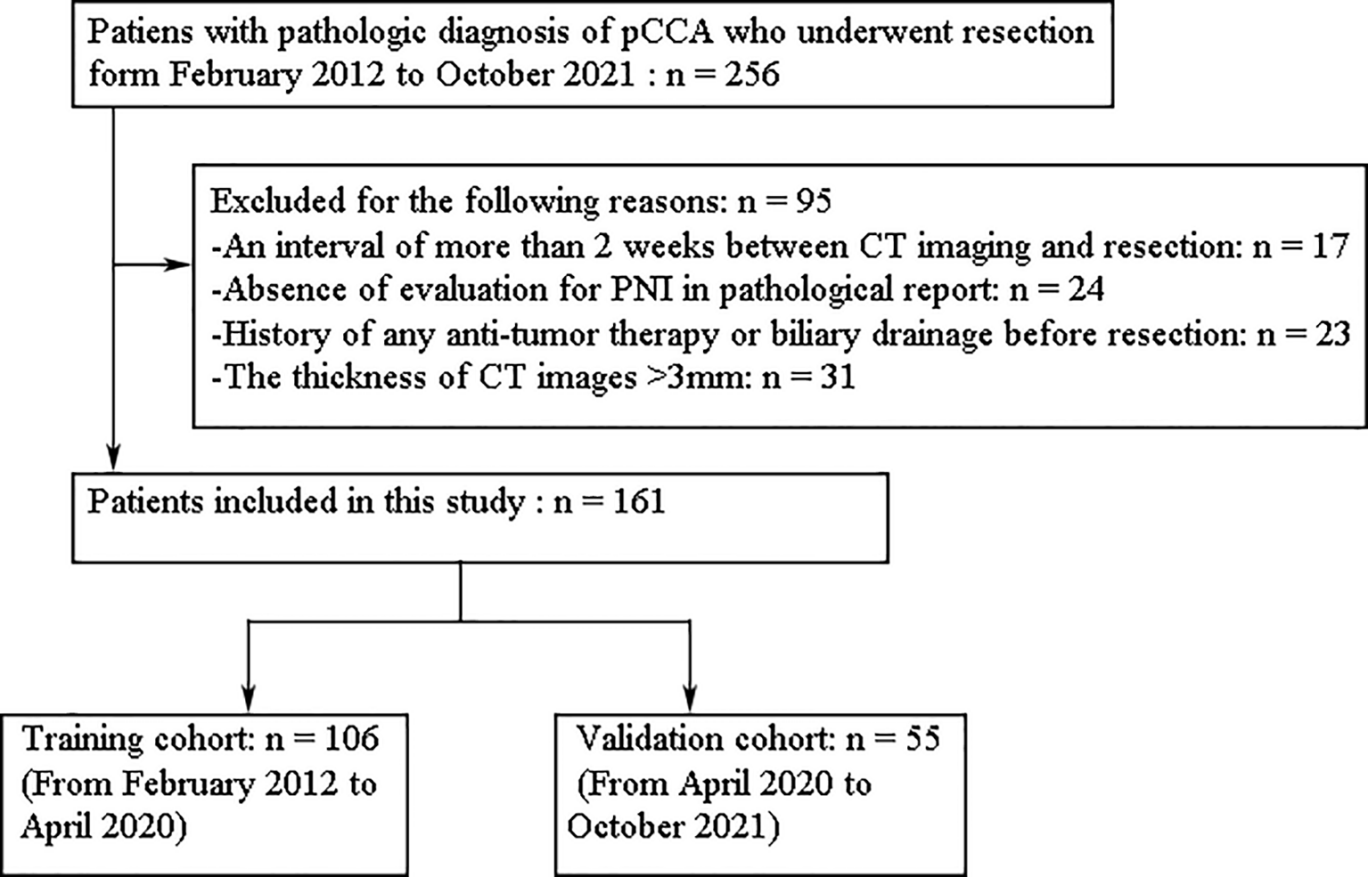
The flowchart of patient selection in this study.

From February 2012 to October 2021, we reviewed clinicopathological characteristics and CT images of 256 consecutive patients with pCCA. Finally, a total of 161 patients were included in the present study based on the inclusion criteria and exclusion criteria. The flowchart of patient selection was presented in **Figure 1**. PNI is defined as the appearance of tumor cells along the nerves and/or within the epineural, perineural, and endoneurial places of the neuronal sheath, with cancer cells surrounding at least 33% of the nerves (26). The inclusion criteria were (1) pathologically diagnosed with pCCA and PNI status evaluation available, (2) curative or palliative resection, and (3) contrast-enhanced CT performed within 2 weeks prior to resection. The exclusion criteria were (1) absence of evaluation for PNI in the pathological report, (2) history of any anti-tumor therapy or biliary drainage before resection, and (3) the thickness of CT images >3mm. According to the order of diagnosis time, we divided the patients into the training cohort (n = 106) and the validation cohort (n = 55). The training cohort was used to select robust radiomics features and construct models, and the internal validation was performed in the validation cohort.

Baseline clinical features were obtained by reviewing the electronic medical charts, including age, sex, symptom, total bilirubin (TBIL), direct bilirubin (DBIL), aspartate aminotransferase (AST), alanine aminotransferase (ALT), glutamyl transpeptidase (GGT), albumin (ALB), prothrombin time (PT), Child-Pugh grade, CA19-9, CEA, CA125, Bismuth classification, clinical T stage (cT), and clinical N stage (cN).

### CT Image Acquisition and Analysis

Contrast-enhanced CT examination of the abdomen was performed in all patients by using a 256-slice CT scanner (Revolution CT, GE Healthcare, United States) or a 320-MDCT scanner (Aquilion ONE, Otawara, Japan). The CT scan sequence included plain scan sequence, arterial phase sequence (AP), and portal venous phase sequence (VP). The intravenous contrast agent (Ultravist 370, Bayer Schering Pharma, Germany) was infused through the antecubital vein at a rate of 3.0–4.0 mL/s (1.5 ml/kg) using a pump injector. AP and VP contrast-enhanced CT images were achieved after a post-injection delay of 20-30 s and 55-70 s, respectively. The parameters of image acquisition are as follows (1) Revolution CT: tube voltage: 120 kv; tube current range: 50-500 mA, pitch: 0.992:1, rotation time: 0.5 s, detector width: 80 mm; reconstruction algorithm: STAND; scan slice thickness: 5 mm; reconstructed section thickness: 0.625 mm. (2) Aquilion ONE: tube voltage: 120 kv; tube current: 350 mA, rotation time: 0.5 s, scan slice thickness: 5 mm, reconstructed section thickness: 2 mm.

Two radiologists with more than 5 years of experience in abdominal imaging who were blinded to pathologic details reviewed CT images and evaluated the following features: Bismuth classification, cT stage, and cN stage. Any discrepancies between the readers were resolved by consultation.

### ROI Segmentation and Feature Extraction

The 3D region of interest (ROI) segmentation was performed using the open-source image analysis software 3D Slicer 4.13.0 (https://www.slicer.org/). Venous phase (VP) images were previously reported as the best phase for pCCA visualization and therefore were used for the segmentation of ROI (27, 28). To evaluate interobserver reliability by calculating the intraclass correlation coefficient (ICC), we randomly chose 50 patients, and segmentation of ROI was performed by one abdominal radiologist (reader 1) with 7 years of experience and another abdominal radiologist (reader 2) with 10 years of experience, who were all blinded to pathologic data. The remaining ROI segmentation was finished by reader 1 and was examined by reader 2. If the ROI was questioned, it would be re-segmented after the two agree.

Before radiomics features extraction, the images were preprocessed to reduce the effect of different scanning schemes or devices on the quantitative radiomics features. First, all images were resampled into 3 × 3 × 3 mm^3^ voxels. In addition, the gray values were discretized using 25 bin width. The radiomic features were then extracted from ROI drawn by reader 1 using the 3D slicer software with an extended plug-in called “PyRadiomics package” (https://www.radiomics.io/pyradiomics.html) (18, 29).

### Feature Selection

To identify robust and reliable radiomics features, feature selection was performed in the following three steps. First, features with ICC greater than 0.75 were included in further feature selection. Furthermore, the correlation analysis was performed to exclude redundant features (30); for each highly correlated feature pair (Pearson correlation coefficient > 0.9), the feature with a higher average absolute correlation was removed. Finally, to prevent overfitting or selection bias, the least absolute shrinkage and selection operator (LASSO) regression with tenfold cross-validation was applied to select the most significant features for PNI. After feature selection, the remaining radiomics features were standardized with the z-score for further analysis (31).

### Models Construction and Evaluation

The radiomics signature was built based on the selected features, and the corresponding radiomics score was calculated for each patient. Based on the selected features, the radiomics model was established by a multivariate logistic regression algorithm.

Laboratory variables were categorized based on normal reference ranges, including those for TBIL (≤25 or >25 umol/L), DBIL (≤10 or >10 umol/L), AST (≤40 or >40 U/mL), ALT (≤50 or >50 U/mL), ALB (≤36 or >36 g/L), CA19-9 (≤40 or >40 U/mL), CEA (≤5 or >5 ng/ml), and CA125 (≤35 or >35 U/mL). The baseline clinical features were subsequently compared by univariate logistic analysis. Statistically significant variables (*p* < 0.05) were included in the multivariable logistic regression analysis, and the clinical model was established. In addition, a clinical-radiomics model was developed integrating the radiomics signature and the independent clinical risk factors.

The performance of different models was measured by AUC, and pairwise ROC comparisons between models were tested using the Delong method. Finally, the model with the best performance was visualized as a nomogram, and its calibration and clinical usefulness were assessed.

### Statistical Analysis

Continuous variables (age) were expressed as mean ± standard deviation (SD). Continuous variables were compared by using the Student t-test or Mann-Whitney U test. Categorical variables were compared by using the χ^2^ test or Fisher exact test. Feature selection, model construction, and performance evaluation were performed on the R software package (version 4.0.3). A two-tailed *p* value < 0.05 was considered statistically significant.

## Results

### Patient Characteristics

**Figure 2:**
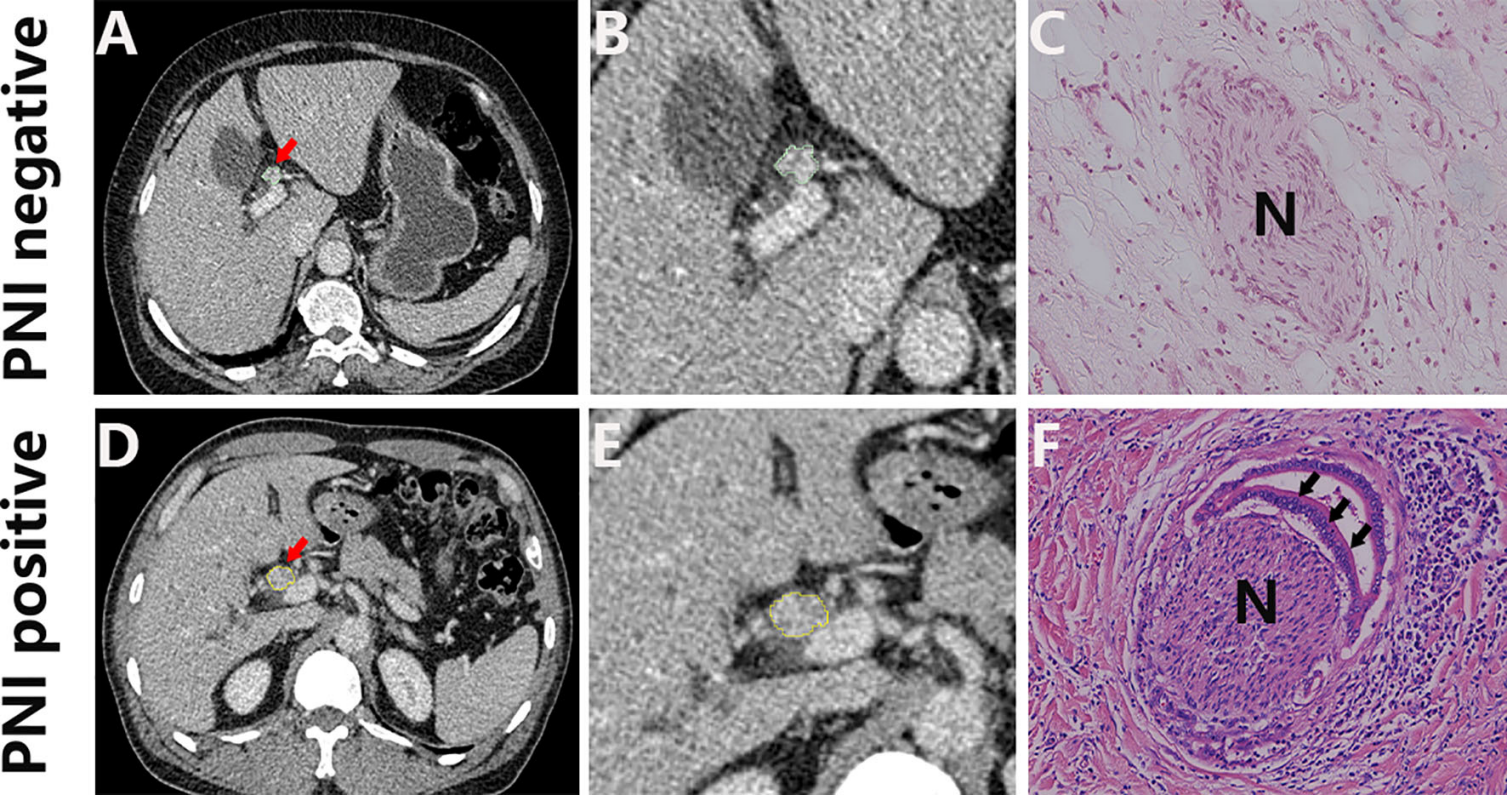
Representative CT images (**A, D**; arrow) and the corresponding cropped images **(B, E)**, and the corresponding histology of PNI negative **(C)** and PNI positive tumors **(F)**. H&E, hematoxylin and eosin, ×150.

A total of 161 patients were enrolled in this study based on the inclusion criteria and exclusion criteria. According to the order of diagnosis time, the patients were divided into the training cohort (n = 106) and the validation cohort (n = 55). The histologic type of all patients is adenocarcinoma, and the baseline clinical characteristics of two cohorts are summarized in **Table 1**. An example of cases with or without PNI is listed in **Figure 2**,

**Table 1:** Characteristics of patients in the training and validation cohort.

Characteristic	Training cohort (n = 106)			Validation cohort (n = 55)		
	PNI negative	PNI positive	*P* Value	PNI negative	PNI positive	*P* Value
**Age (years)**	60.4±10.7	60.6±9.9	0.920	65.6±6.4	62.4±8.4	0.152
**Sex**			0.078			0.022
Female	16	31		9	11	
Male	11	48		5	30	
**symptom**			0.473			0.927
Jaundice	14	44		8	24	
Abdominal malaise	5	20		3	7	
Both	8	15		3	10	
**TBIL (umol/L)**			> 0.999			0.012
≤25	4	11		4	1	
>25	23	68		10	40	
**DBIL (umol/L)**			0.417			0.047
≤10	3	5		3	1	
>10	24	74		11	40	
**AST (U/L)**			0.755			0.181
≤40	3	12		4	4	
>40	24	67		10	37	
**ALT (U/L)**			0.508			0.638
≤40	2	11		2	4	
>40	25	68		12	37	
**ALB (g/L)**			0.349			0.007
≤40	25	65		7	36	
>40	2	14		7	5	
**Child-Pugh grade**			> 0.999			0.703
A	4	11		3	7	
B	23	68		11	34	
**CA19-9 (U/mL)**			0.392			0.259
≤40	7	13		5	7	
>40	20	66		9	34	
**CEA (ng/mL)**			0.805			> 0.999
≤5	19	58		11	32	
>5	8	21		3	9	
**CA125 (U/mL)**			0.553			> 0.999
≤35	24	64		14	39	
>35	3	15		0	2	
**Bismuth classification**			< 0.001			0.529
I/II	22	24		7	26	
III/IV	5	55		7	15	
**cT stage**			0.446			> 0.999
1/2	22	57		13	36	
3/4	5	22		1	5	
**cN stage**			0.801			> 0.999
0	19	59		9	27	
1/2	8	20		5	14	
**Radiomics score**	0.362±0.320	0.876±0.175	< 0.001	0.301±0.352	0.816±0.295	< 0.001

### Feature Extraction and Selection

The plug-in called the “PyRadiomics package” of 3D slicer software was applied to extract radiomics features from each ROI. The extracted features were reproducible and matched the benchmarks of the image biomarker standardization initiative (IBSI) (32). For each patient, 851 radiomics features were extracted, including 14 shape features, 18 first-order features, 75 texture features, and 744 wavelet features. The details of extracted radiomics features were presented in Supplemental **Appendix 1**; **Table S1**.

**Figure 3:**
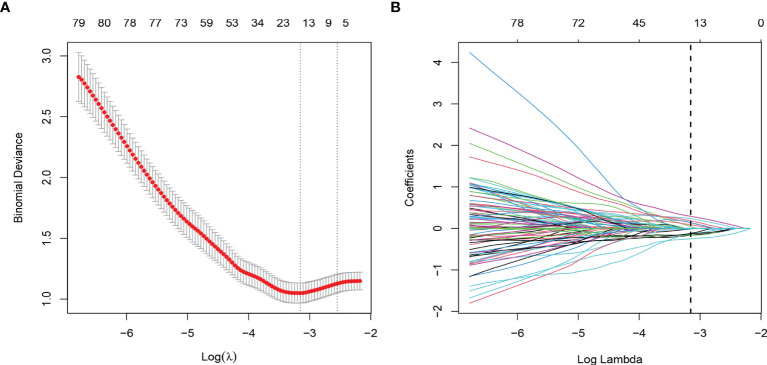
Radiomic feature selection by using the least absolute shrinkage and selection operator (LASSO) logistic regression. **(A)** The selection of tuning parameter (λ) in the LASSO model used 10-fold cross-validation *via* minimum criteria. The AUC curve was plotted versus log (λ). **(B)** LASSO coefficient profiles of the radiomics features. A vertical line was plotted at the optimal λ value, which resulted in 15 features with nonzero coefficients.

**Figure 4:**
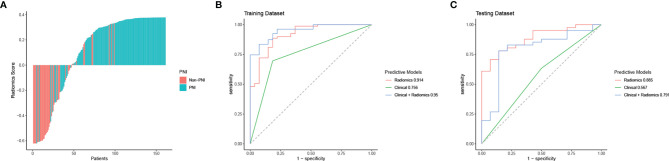
Radiomics score, ROC curve analysis of all models in the training dataset and the testing dataset. **(A)** Waterfall plot for distribution of radiomics scores for each patient. **(B)** ROC curves of all models for predicting PNI in the training dataset. **(C)** ROC curves of all models for predicting PNI in the testing dataset.

Through ICC analysis and the correlation analysis, 487 radiomics features were excluded, and the remaining 364 stable features were considered for subsequent analysis. Finally, based on LASSO regression, 15 PNI status related features with nonzero coefficients were selected, and the radiomics signature was constructed. The feature selection process of LASSO is illustrated in **Figure 3**. The corresponding radiomics score was calculated for each patient and was shown in **Figure 4A**. The formula of the radiomics score and the details of the selected radiomics features were presented in **Supplemental Appendix 2**; **Table S2**.

### Models Construction and Evaluation

Among all baseline clinical features, only Bismuth classification was identified as a significant predictor for PNI by univariate analysis. So the clinical model was built based on the Bismuth classification. Detailed univariate analysis results were shown in **Table 2**. In addition, by using a multivariate logistic regression algorithm, the radiomics model was established based on the radiomics signature and the combined model was developed by integrating the radiomics signature and Bismuth classification.

**Table 2:** Univariate logistic regression in the training cohort.

Variable	Odd Ratio	*P* value
**Age**	1.002 (0.958, 1.046)	0.916
**Sex**	0.444 (0.178, 1.072)	0.074
**Symptom**	1.273 (0.403, 4.019)	0.681
**TBIL**	0.930 (0.286, 3.612)	0.909
**DBIL**	0.541 (0.123, 2.790)	0.423
**AST**	1.433 (0.412, 6.679)	0.601
**ALT**	2.022 (0.497, 13.657)	0.381
**ALB**	0.371 (0.056, 1.458)	0.211
**Child Pugh grade**	1.075 (0.277, 3.498)	0.909
**CA19-9**	0.563 (0.201, 1.671)	0.281
**CEA**	1.163 (0.426, 2.993)	0.759
**CA125**	0.533 (0.116, 1.797)	0.353
**Bismuth classification**	10.083 (3.651, 33.024)	< 0.001
**cT**	1.698 (0.606, 5.562)	0.340
**cN**	0.805 (0.311, 2.207)	0.661

**Figure 5:**
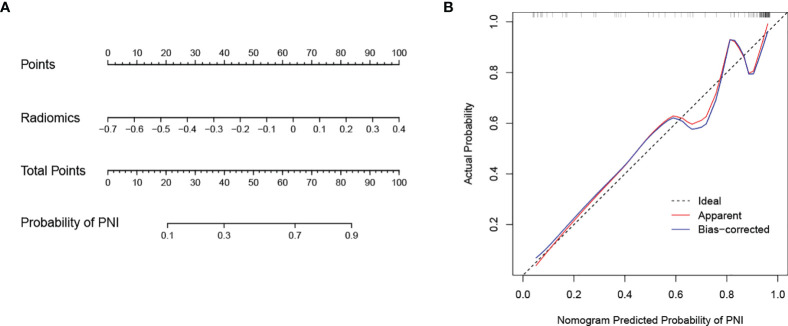
Nomogram developed with the radiomics model and calibration curves of the nomogram. **(A)** The developed radiomics nomogram to predict PNI in patients with pCCA. **(B)** Calibration curves of the nomogram. The x-axis and the y-axis show the nomogram predicted probabilities of PNI and the actual probabilities, respectively. The diagonal gray line presents a perfect prediction, and the red solid line presents the predictive performance of the nomogram. Better prediction is indicated by a closer fit of the red solid line to the diagonal gray line.

In the training cohort, the AUC values of the clinical model, the radiomics model, and the combined model were 0.756 (95% CI 0.665-0.846), 0.914 (95% CI 0.853-0.976), and 0.950 (95% CI 0.912-0.988), respectively. In the validation cohort, the AUC values of three corresponding models were 0.567 (95% CI 0.412-0.722), 0.885 (95% CI 0.797-0.974), and 0.791 (95% CI 0.642-0.940), respectively. Though the combined model showed a higher predictive capability in the training cohort, the AUC values of the combined model and the clinical model were significantly less than the radiomics model. Therefore, the radiomics model was chosen as the final model and presented as a nomogram. The specific performances of models are shown in **Table 3**. ROC curves of the three models are illustrated in **Figure 4**, and the nomogram is presented in **Figure 5A**.

**Table 3:** Performances of models for PNI prediction.

Model	Training cohort				Validation cohort			
	Sensitivity (%)	Specificity (%)	Accuracy (%)	AUC (95%CI)	Sensitivity (%)	Specificity (%)	Accuracy (%)	AUC (95%CI)
**Clinical**	0.696	0.815	0.726	0.756 (0.665-0.846)	0.634	0.500	0.600	0.567 (0.412-0.722)
**Radiomics**	0.886	0.815	0.868	0.914 (0.853-0.976)	0.829	0.643	0.782	0.885 (0.797-0.974)
**Combined**	0.835	0.926	0.858	0.950 (0.912-0.988)	0.780	0.857	0.800	0.791 (0.642-0.939)

**Figure 6:**
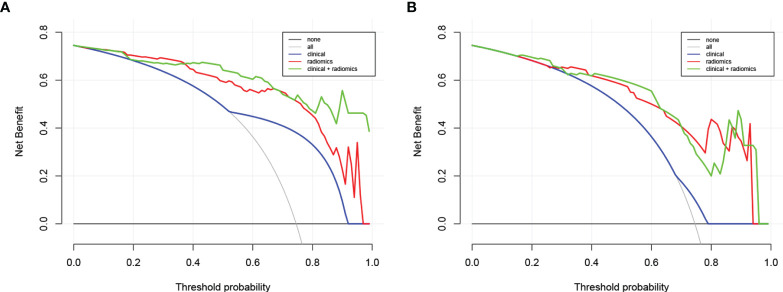
Decision curve analysis for the combined model, radiomics model, and clinical model in the training dataset **(A)** and in the testing dataset **(B)**. The y-axis represents the net benefit. The gray line represents the assumption that all patients were confirmed with PNI; however, the black line is the opposite. The blue line represents the clinical model. The red line represents the radiomics model. The green line represents the combined model.

The calibration curve of the nomogram is presented in **Figure 5B** and indicated good agreement between predictive probability and actual PNI status. The decision curve analysis (DCA) for the radiomics model, the clinical model, and the combined model are presented in **Figure 6**. The DCA demonstrated good performance of the radiomics model in terms of clinical decision-making. In addition, the radiomics model and the combined model had a similar clinical application value, and they can provide a better net benefit than the clinical model.

## Discussion

The present study was conducted to probe the value of CT radiomics in determining PNI of pCCA. Our results revealed that the CT-radiomics nomogram based on the radiomics signature had a good performance in detecting PNI. The calibration curve and the DCA demonstrated that the nomogram was well calibrated and had significantly more clinical net benefits. This is the first CT-based radiomics model to noninvasively predict PNI of pCCA.

Several conventional imaging methods have been reported to diagnose PNI. Asayama et al. found that enhancement of greater than two-thirds of the primary lesion on delayed phase images was correlated with PNI in 32 patients with intrahepatic cholangiocarcinoma (ICC) (33). But this conclusion was not confirmed in another study, in which soft-tissue infiltration along the celiac plexus on MDCT was regarded as an indicator of PNI in 20 ICC patients (34). In addition, several studies have shown the value of 3D volume-rendered MDCT imaging in diagnosing PNI of pancreatic adenocarcinoma (35, 36). These studies all have the important limitation of a small sample size. On the other hand, it is difficult to confirm PNI through visual evaluation and simple quantitative methods (22).

As an emerging field, radiomics has achieved satisfactory abilities in the clinical diagnosis and treatment of malignant tumors. Previous studies have reported radiomics models with good performance in predicting the lymph node metastasis, preoperative staging, and postoperative recurrence of pCCA (27, 37, 38). In addition, many studies have indicated the diagnostic value of radiomics in detecting PNI of specific tumors including colorectal cancer, head and neck squamous cell carcinoma, and ICC (21, 24, 25, 39). Huang et al. reported an MRI-based radiomics model with the AUC value of 1 to predict PNI in patients with extrahepatic cholangiocarcinoma (22). But their study included two subtypes of CCA, and the sample size was only 101 patients. In the present study, we enrolled 161 patients with pCCA, and establish a CT-based radiomics model. The radiomics model achieved satisfactory performance in both two cohorts, with the AUCs of 0.914 and 0.885, respectively. Of note, the selected radiomics features in our study were all wavelet-based features with image conversion using a wavelet filter, which demonstrated wavelet-based features were more sensitive to PNI of the patients. Previous studies reported that wavelet-based features furnished more directional information than conventional features, which played an important role in building radiomics signatures (40). Furthermore, we developed a CT-radiomics nomogram for noninvasive prediction of PNI in pCCA. The CT-radiomics nomogram provides a simple and convenient tool for noninvasive prediction of PNI in pCCA, which may be helpful to improve patient prognostic stratification and facilitate optimized and individualized treatment strategies.

Among all baseline clinical features, we did not find the independent predictor for PNI of pCCA. That may be because we didn’t include enough clinical features or data bias. A study by Li et al. demonstrated that the depth of tumor invasion was correlated significantly with PNI of pCCA (41). Besides, some studies reported several clinicopathological factors related to PNI of other specific tumors including tumor markers, tumor stage, and tumor differentiation grade (19–21, 42). Of note, our study aimed to develop a noninvasive prediction model before surgery resection, hence pathological factors were not considered in our study. Moreover, clinical factors are indispensable for the prediction of tumors, and more efforts should be made to explore the clinical factors associated with PNI of pCCA.

Our study has several limitations. First, there may exist data bias due to the retrospective design of this study. Second, this study was conducted as a single-center study, and a future multicenter study is necessary to validate and improve the performance of our model. In addition, further study should be performed to reveal the relationship between the selected radiomics features and clinical features or genomic features, and our radiomics model should be further refined by incorporating other multi-omics features.

### Conclusion

We constructed a radiomics model with good performance based on selected radiomics features to noninvasively predict PNI of pCCA. The prediction model has potential prognostic value to stratify patients and may provide a reference for individualized treatment of pCCA patients.

## Data Availability Statement

The raw data supporting the conclusions of this article will be made available by the authors, without undue reservation.

## Ethics Statement

The studies involving human participants were reviewed and approved by the Ethical Committee of the First Affiliated Hospital of Zhengzhou University (2021-KY-0778-001). Written informed consent for participation was not required for this study in accordance with the national legislation and the institutional requirements.

## Author Contributions

PZ and PL designed this work. PZ, XL, HW, NL, YZ, WH and YC integrated and analyzed the data. PZ, ZL and XL wrote this manuscript. PZ, PL, ZL and JG edited and revised the manuscript. All authors approved this manuscript.All authors contributed to the article and approved the submitted version. All authors approved this manuscript. All authors contributed to the article and approved the submitted version.

## Funding

This study was supported by the Key Scientific Research Project of Higher Education in Henan Province (No.22A320057).

## Conflict of Interest

The authors declare that the research was conducted in the absence of any commercial or financial relationships that could be construed as a potential conflict of interest.

## Publisher’s Note

All claims expressed in this article are solely those of the authors and do not necessarily represent those of their affiliated organizations, or those of the publisher, the editors and the reviewers. Any product that may be evaluated in this article, or claim that may be made by its manufacturer, is not guaranteed or endorsed by the publisher.
