# Supplementary material for: CT-Based Radiomics Analysis for Noninvasive Prediction of Perineural Invasion of Perihilar Cholangiocarcinoma
Source: Front Oncol. 2022 Jun 20;12:900478. doi: 10.3389/fonc.2022.900478 (PMC9252420; doi:10.3389/fonc.2022.900478)
Supplement: Supplementary file 1 [file DataSheet_1.docx]

**Appendix 1; Table S1 Detailed information of extracted radiomics features.**

| **feature type** | **feature name** |
| --- | --- |
| Shape features (n=14) | (1) Elongation; (2) Flatness; (3) LeastAxisLength;  (4) MajorAxisLength; (5) Maximum2DDiameterColumn;  (6) Maximum2DDiameterRow;  (7) Maximum2DDiameterSlice;  (8) Maximum3DDiameter; (9) MeshVolume;  (10) MinorAxisLength; (11) Sphericity; (12) SurfaceArea; (13) SurfaceVolumeRatio; (14) VoxelVolume |
| First-order features (n=18) | (1)10Percentile; (2) 90Percentile; (3) Energy; (4) Entropy;  (5) InterquartileRange; (6) Kurtosis; (7) Maximum;  (8) MeanAbsoluteDeviation; (9) Mean; (10) Median;  (11) Minimum; (12) Range;  (13) RobustMeanAbsoluteDeviation; (14) RootMeanSquared; (15) Skewness; (16) TotalEnergy; (17) Uniformity;  (18) Variance |
| Gray Level Co-occurrence Matrix (GLCM) features (n=24) | (1) Autocorrelation; (2) JointAverage; (3) ClusterProminence; (4) ClusterShade; (5) ClusterTendency; (6) Contrast;  (7) Correlation; (8) DifferenceAverage;  (9) DifferenceEntropy; (10) DifferenceVariance;  (11) JointEnergy; (12) JointEntropy; (13) Imc1; (14) Imc2;  (15) Idm; (16) Idmn; (17) Id; (18) Idn; (19) InverseVariance; (20) MaximumProbability; (21) SumEntropy;  (22) SumSquares; (23) MCC; (24) SumAverage |
| Gray Level Size Zone Matrix  (GLSZM) features (n=16) | (1) GrayLevelNonUniformity;  (2) GrayLevelNonUniformityNormalized;  (3) GrayLevelVariance; (4) HighGrayLevelZoneEmphasis;  (5) LargeAreaEmphasis;  (6) LargeAreaHighGrayLevelEmphasis;  (7) LargeAreaLowGrayLevelEmphasis;  (8) LowGrayLevelZoneEmphasis;  (9) SizeZoneNonUniformity;  (10) SizeZoneNonUniformityNormalized;  (11) SmallAreaEmphasis;  (12) SmallAreaHighGrayLevelEmphasis;  (13) SmallAreaLowGrayLevelEmphasis; (14) ZoneEntropy; (15) ZonePercentage; (16) ZoneVariance |
| Gray Level Run Length Matrix (GLRLM) features (n=16) | (1) GrayLevelNonUniformity;  (2) GrayLevelNonUniformityNormalized;  (3) GrayLevelVariance  (4) HighGrayLevelRunEmphasis; (5) LongRunEmphasis;  (6) LongRunHighGrayLevelEmphasis;  (7) LongRunLowGrayLevelEmphasis;  (8) LowGrayLevelRunEmphasis; (9) RunEntropy;  (10) RunLengthNonUniformity;  (11) RunLengthNonUniformityNormalized;  (12) RunPercentage; (13) RunVariance;  (14) ShortRunEmphasis;  (15) ShortRunHighGrayLevelEmphasis;  (16) ShortRunLowGrayLevelEmphasis |
| Gray Level Dependence Matrix  (GLDM) Features (n=14) | (1) DependenceEntropy; (2) DependenceNonUniformity;  (3) DependenceNonUniformityNormalized;  (4) DependenceVariance; (5) GrayLevelNonUniformity;  (6) GrayLevelVariance; (7) HighGrayLevelEmphasis;  (8) LargeDependenceEmphasis;  (9) LargeDependenceHighGrayLevelEmphasis;  (10) LargeDependenceLowGrayLevelEmphasis;  (11) LowGrayLevelEmphasis;  (12) SmallDependenceEmphasis;  (13) SmallDependenceHighGrayLevelEmphasis;  (14) SmallDependenceLowGrayLevelEmphasis |
| Neighborhood gray-tone difference matrix (NGTDM) features  (n=5) | (1) Busyness；(2) Coarseness；(3) Complexity；  (4) Contrast; (5) Strength |
| Wavelet features (n=744) | (1) LLL; (2) LLH; (3) LHL; (4) LHH;  (5) HLL; (6) HLH; (7) HHL; (8) HHH |
|  | For each decomposition, First-order features, GLCM，GLSZM，GLRLM，GLDM were extracted on the above 8 filtered images. Therefore, the total number of wavelet features could be calculated as (18+24+16+16+14+5) ×8= 744. |

The feature pool comprised 18 original first-order histogram features, 14 original shape features, 75 original textural features, including 24 gray-level cooccurrence matrix (GLCM) features, 14 gray-level dependence matrix (GLDM) features, 16 gray-level run-length matrix (GLRLM) features, 16 gray-level size zone matrix (GLSZM), 5 neighborhood gray-tone difference matrix (NGTDM) features, and 744 high-order wavelet features.

In total, 851 radiomics features 14 shape features + (75 textural features+18 first-order features) × (1 original image  + 8 wavelet filtered images) were calculated for each patient.

**Appendix 2; Table S2 Detailed information of the selected radiomics features**

| **Feature** | **Coefficient** |
| --- | --- |
| Intercept | \| 2.090243242 \| \| --- \| |
| wavelet.LLH_firstorder_Mean | 1.412411423 |
| wavelet.LLH_glcm_InverseVariance | -0.404638884 |
| wavelet.LHL_firstorder_InterquartileRange | 0.893849975 |
| wavelet.LHL_firstorder_Skewness | -1.068160004 |
| wavelet.LHL_glcm_Correlation | 1.976276063 |
| wavelet.HLL_gldm_LargeDependenceHighGrayLevelEmphasis | -0.879405642 |
| wavelet.HLL_glszm_SmallAreaEmphasis | 0.070010087 |
| wavelet.HLH_firstorder_Skewness | 0.860498131 |
| wavelet.HLH_glrlm_GrayLevelNonUniformityNormalized | 1.321332361 |
| wavelet.HHL_firstorder_Kurtosis | -0.206672244 |
| wavelet.HHL_glcm_Idn | -0.175628084 |
| wavelet.HHL_glszm_SizeZoneNonUniformityNormalized | -1.408525199 |
| wavelet.HHL_glszm_SmallAreaLowGrayLevelEmphasis | 0.783884345 |
| wavelet.HHH_firstorder_Mean | -1.528793756 |
| wavelet.LLL_gldm_LargeDependenceLowGrayLevelEmphasis | 0.552901756 |

Radiomics score = 2.090243242 + wavelet.LLH_firstorder_Mean × 1.412411423

+ wavelet.LLH_glcm_InverseVariance × (-0.404638884)

+ wavelet.LHL_firstorder_InterquartileRange × 0.893849975

+ wavelet.LHL_firstorder_Skewness × (-1.068160004)

+ wavelet.LHL_glcm_Correlation × 1.976276063

+ wavelet.HLL_gldm_LargeDependenceHighGrayLevelEmphasis × (-0.879405642)

+ wavelet.HLL_glszm_SmallAreaEmphasis × 0.070010087

+ wavelet.HLH_firstorder_Skewness × 0.860498131

+ wavelet.HLH_glrlm_GrayLevelNonUniformityNormalized × 1.321332361

+ wavelet.HHL_firstorder_Kurtosis × (-0.206672244)

+ wavelet.HHL_glcm_Idn × (-0.175628084)

+ wavelet.HHL_glszm_SizeZoneNonUniformityNormalized × (-1.408525199)

+ wavelet.HHL_glszm_SmallAreaLowGrayLevelEmphasis × 0.783884345

+ wavelet.HHH_firstorder_Mean × (-1.528793756)

+ wavelet.LLL_gldm_LargeDependenceLowGrayLevelEmphasis × 0.552901756
